# Supplementary material for: Motivated reasoning in the field: polarization of prose, precedent, and policy in U.S. Circuit Courts, 1891–2013
Source: PLoS One. 2025 Mar 3;20(3):e0318790. doi: 10.1371/journal.pone.0318790 (PMC11875365; doi:10.1371/journal.pone.0318790)
Supplement: S1 Text — (PDF) [file pone.0318790.s001.pdf]

Supporting Information for Motivated Reasoning in the Field: Polarization of Prose, Precedent, and Policy in U.S. Circuit Courts, 1891-2013

Training

Text Classification

We implement fine-tuning on three popular transformer-based pre-trained models and use a simple average ensemble of predictions as the final predictions of texts on political affiliations of judges. The first model we use is DistilBERT [1], a smaller version of the BERT model designed to overcome the slow training problem of BERT [2] due to the large model size while obtaining similar performance as BERT. Secondly, we use two improved version of BERT, XLnet [3] and twitter-RoBERTa [4] that are trained on larger corpus and with improved architecture than the original BERT model.

For fine-tuning, we used the Python package `transformer` and accessed pre-trained models from Huggingface.co, a collaborative open-source platform for model sharing. The distilbert-base-uncased-finetuned-sst-2-english model was fine-tuned using default parameters over five epochs on 70% of a 10% sample (comprising 22,922 opinions), with the remainder serving as the test set. The xlnet-base-cased and twitter-roberta-base-sentiment-latest models were trained on 70% of a 5% sample for five epochs with a learning rate of 2e-5, other parameters being default, due to computational limitations. Post fine-tuning, these models were applied to the entire sample for political party predictions.

Overall, three models exhibited comparable results, consistently achieving a prediction accuracy around 0.7, shown in Table 1. Altering the number of epochs from two to eight did not significantly impact the outcomes, as we consistently employed the best model for predictions.

Table 1. Model Performance Metrics, Text Classification

Model	Training Loss	Validation Loss	Accuracy	N
DistilBERT	0.5013	0.5481	0.707588	22,292
twitter-RoBERTa	0.4897	0.5960	0.700084	11,146
XLnet	0.4740	0.5926	0.698619	11,146

Citation Classification

We first use a grid search method with K-fold cross validation to tune the parameters used in different algorithms (a list of commonly used algorithms) in order to maximize the evaluation metric of that algorithm (here we used the AUC score). Then we use a voting ensemble method based on the best estimator of each model to average results obtained from the set of algorithms. The analysis is done using Python packages `scikit-learn` and `xgboost`. After training, we apply the ensemble model on full sample.

For each algorithm, we allow the algorithm to search among a set of possible parameters to optimize the prediction, as in [5]:

- Elastic Net. A10-fold cross validation is added to the algorithm to choose the optimal mixing parameter of LASSO and ridge regression among a vector of possible choices: $[0.1, 0.15, 0.5, 0.7, 0.95, 0.99, 1]$.
- Decision Tree. We use a 10-fold cross validation to choose the optimal minimal samples per leaf among a vector of possible choices: $[1, 5, 10, 20, 50, 100, 150, 500, 1000]$.
- Random Forest. We use a 10-fold cross validation to choose the optimal minimal samples per leaf among a vector of possible choices: $[5, 10, 20, 50, 100, 200, 500, 1000]$.
- XGBoost, by [6]. We use a 10-fold cross validation to choose the optimal maximum number of leafs among a vector of possible choices: $[3, 5, 10, 20, 50, 100, 200, 500, 1000]$.
- K-Nearest Neighbors. We use a 10-fold cross validation to choose the optimal number of neighbors among a vector of possible choices: $[20, 50, 100, 200, 300, 500]$.

Overall, the voting ensemble is as good as every individual algorithms, and the accuracy is around 0.60, as shown in Table 2.

Table 2. Model Performance Metrics, Citation Classification

Algorithm	F1 Score	Accuracy	N
Elastic Net	0.5621	0.5821	192,758
Regression Tree	0.5651	0.5764	192,758
Random Forest	0.5865	0.5974	192,758
XGBoost	0.5763	0.5868	192,758
K-Nearest Neighbors	0.5682	0.5884	192,758
Voting Ensemble	0.5797	0.5946	192,758

Polarization across Time

In this section, we re-examined the patterns presented in Figure 1 of the main paper, employing a linear regression model with fixed effects for Circuit Court and Legal Issue. This analysis aimed to assess polarization across three dimensions: prose, precedent, and policy. As shown in Figure 1, a marked increase in textual polarization is observed starting from the 1970s, indicating a shift towards more politically charged language in judicial opinions. In contrast, precedent polarization does not show a significant change, reinforcing the notion that language, rather than legal precedents, has become a primary medium for expressing politically motivated reasoning. Furthermore, dissent rates along party lines have been on the rise since the 1970s, suggesting an increasing tendency for judges to vote in accordance with their political affiliations.

Polarization by Experience

To explore the underlying mechanism of behavioral anomalies, we examined if such anomalies diminish with experience. Specifically, we focused on whether anomalies are driven by Type I thinking, which may erode with experience, unlike Type II thinking, like motivated reasoning, which are more reflective and intentional. Using the same linear regression framework, we analyzed how polarization in reasoning varies with judges' experience. Our findings, presented in Table 3, reveal that polarization in prose remains largely unchanged with experience, except for a notable increase among judges with 15 to 25 years of experience. These results suggest that while judges' experience do not significantly impact polarization in their textual content, their selection of precedents becomes a bit more polarized in the middle of their careers. This finding is particularly striking given the overall increase in textual polarization over the years, suggesting that this trend might not be primarily driven by the accumulation of judicial experience. Further research is necessary to fully understand these dynamics and the factors influencing them. These patterns, where prose polarization is mostly unaffected by experience, suggest that the behavioral anomalies are driven by Type II thinking, being more reflective and intentional in nature.

Polarization during Vacancies

As noted by [7], since the era of President Eisenhower, there has been a growing trend for presidents to prefer individuals from federal courts as potential Supreme Court

candidates. This preference may be attributed to the clearer ideological traceability of federal judges compared to candidates from other backgrounds. Since President Ford's nomination of Justice John G. Roberts, approximately 73% of the nominees have been Circuit Court judges. In light of this trend, our study focuses on all Supreme Court vacancies from 1975 to 2013. We consider the vacancy period, plus the six months preceding it, as our sample timeframe.

Following the approach of [8] for defining vacancies and contenders, we identify the start of a vacancy as the date a justice first informs the president of their intention to step down. The vacancy period ends when the Senate confirms the nomination. We define contenders as judges included in the president's shortlist for each vacancy, based on the criteria established by [7]. Our analytical specification for examining the influence of promotion incentives on judicial polarization is outlined below:

$$Y_{it} = \alpha + \beta Vacancy_t + \gamma Contender_i + \delta Vacancy_t \times Contender_i + \boldsymbol{\eta}' \boldsymbol{Z}_{it} + \varepsilon_{it} \quad (1)$$

where Y_{it} is the polarization outcome (e.g. dissent rate), and $\boldsymbol{Z}_{it}$ are Circuit × Year and legal-issue fixed effects. We estimate the equation using OLS with robust standard errors clustered by individual judge. The coefficient of primary interest is δ, which measures the average difference in the polarization outcome, accounting for the fixed effects, for contenders during the periods of judicial vacancies.

Robustness checks

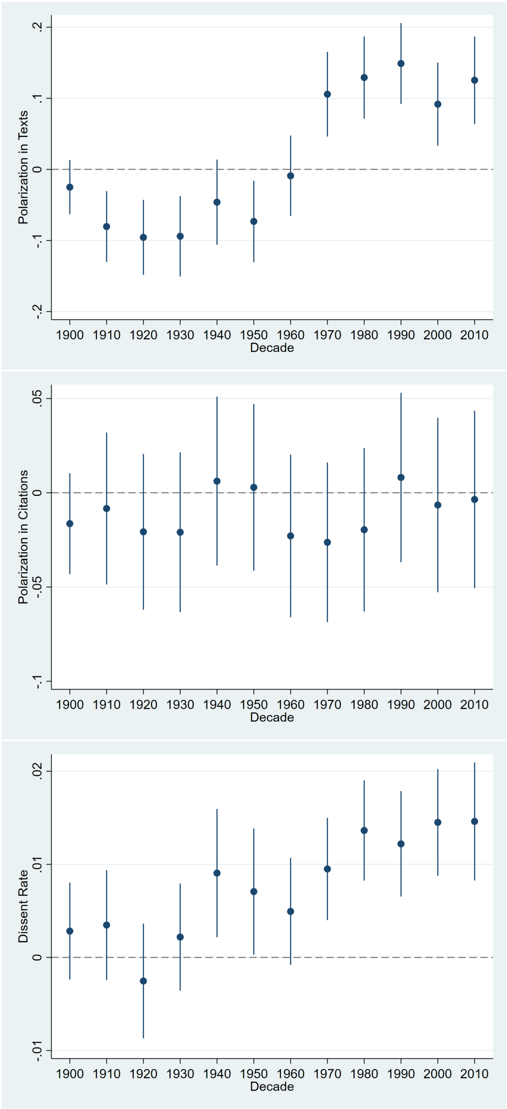

Fig 1. Polarization in prose, Precedent, and Policy across time

Notes: The temporal changes in polarization in texts, citations, and dissent votes. The baseline level is 1890-1900. We control for Circuit and Legal Issue fixed effects. Standard errors clustered at judge level in parentheses.

Table 3. The effect of experience on polarization

	(1) Text	(2) Citation
Age	-0.000	-0.001***
	(0.001)	(0.001)
Experience $\in [5, 10)$	-0.000	0.004
	(0.005)	(0.003)
Experience $\in [10, 15)$	-0.008	-0.001
	(0.010)	(0.006)
Experience $\in [15, 20)$	-0.002	0.017*
	(0.016)	(0.009)
Experience $\in [20, 25)$	-0.005	0.025*
	(0.021)	(0.013)
Experience $\in [25, 30)$	-0.025	0.010
	(0.027)	(0.017)
Experience $\in [30, 35)$	-0.030	-0.007
	(0.033)	(0.022)
Experience $\in [35, 55)$	-0.039	-0.018
	(0.042)	(0.032)
Observations	312930	271059
R^2	0.334	0.117
Circuit × Year FE	✓	✓
Legal Issue FE	✓	✓

Notes: The baseline level is Experience $\in [0, 5)$ years. Standard errors clustered at judge level in parentheses. $*p < .1, **p < 0.05, ***p < .01$.

Table 4. Polarization in Divided Panels (Dropping Court 2, 8, 9, and DC)

	(1) Text	(2) Citation	(3) Dissent vote
Divided Panel	-0.034***	-0.041***	0.004***
	(0.007)	(0.005)	(0.001)
Observations	178396	154920	598232
R^2	0.306	0.123	0.011
Circuit × Year FE	✓	✓	✓
Legal Issue FE	✓	✓	✓

Notes: This table shows how judges on a divided panel would exhibit polarization in prose, precedent, and policy. The unit of observation for Column (1) and (2) is at the opinion level, and Column (3) is at the vote level. Every case has three votes from three judges sitting in a panel and judges are allowed to write concurring or dissent opinions besides the majority opinion for each case. We controlled for Circuit × Year and legal issues fixed effects. Standard errors clustered at judge level in parentheses.* $p < 0.1$,** $p < 0.05$,*** $p < 0.01$

Table 5. Polarization in Divided Panels (Dropping Court 2, 8, 9, and DC)

	Text	Citation	Dissent Vote
	(1)	(2)	(3)
Minority	-0.018**	-0.030***	0.009***
	(0.008)	(0.005)	(0.001)
Observations	129135	112337	426877
R^2	0.295	0.068	0.013
Circuit × Year FE	✓	✓	✓
Legal Issue FE	✓	✓	✓

Notes: Effect of being a minority judge (D of DRR or R of RDD) on the polarization in texts and citations, and the likelihood to cast a dissenting vote, controlling for Circuit × Year and legal issues fixed effects. The unit of observation for Column (1) and (2) is at the opinion level, and Column (3) is at the vote level. Standard errors clustered at judge level in parentheses. The sample is cases with judges from both political parties. $*p < .1, **p < 0.05, ***p < .01$.

Table 6. Polarization in Divided Panels (Senior judges)

	(1)	(2)	(3)
	Text	Citation	Dissent vote
Divided Panel	-0.046***	-0.047***	0.007***
	(0.007)	(0.005)	(0.002)
Observations	100726	87842	327994
R^2	0.356	0.206	0.014
Circuit × Year FE	✓	✓	✓
Legal Issue FE	✓	✓	✓

Notes: This table shows how judges on a divided panel would exhibit polarization in prose, precedent, and policy. The unit of observation for Column (1) and (2) is at the opinion level, and Column (3) is at the vote level. Every case has three votes from three judges sitting in a panel and judges are allowed to write concurring or dissent opinions besides the majority opinion for each case. We controlled for Circuit × Year and legal issues fixed effects. Standard errors clustered at judge level in parentheses.* $p < 0.1$,** $p < 0.05$,*** $p < 0.01$

Table 7. Polarization in Divided Panels (Senior judges)

	Text	Citation	Dissent Vote
	(1)	(2)	(3)
Minority	-0.036***	-0.045***	0.016***
	(0.008)	(0.005)	(0.002)
Observations	100726	87842	327994
R^2	0.354	0.207	0.015
Circuit × Year FE	✓	✓	✓
Legal Issue FE	✓	✓	✓

Notes: Effect of being a minority judge (D of DRR or R of RDD) on the polarization in texts and citations, and the likelihood to cast a dissenting vote, controlling for Circuit × Year and legal issues fixed effects. The unit of observation for Column (1) and (2) is at the opinion level, and Column (3) is at the vote level. Standard errors clustered at judge level in parentheses. The sample is cases with judges from both political parties. $*p < .1, **p < 0.05, ***p < .01$.

References

1. Sanh V, Debut L, Chaumond J, Wolf T. DistilBERT, a distilled version of BERT: smaller, faster, cheaper and lighter. arXiv preprint arXiv:191001108. 2019;.

2. Devlin J, Chang MW, Lee K, Toutanova K. Bert: Pre-training of deep bidirectional transformers for language understanding. arXiv preprint arXiv:181004805. 2018;.

3. Yang Z, Dai Z, Yang Y, Carbonell J, Salakhutdinov RR, Le QV. Xlnet: Generalized autoregressive pretraining for language understanding. Advances in neural information processing systems. 2019;32.

4. Camacho-collados J, Rezaee K, Riahi T, Ushio A, Loureiro D, Antypas D, et al. TweetNLP: Cutting-Edge Natural Language Processing for Social Media. In: Proceedings of the 2022 Conference on Empirical Methods in Natural Language Processing: System Demonstrations. Abu Dhabi, UAE: Association for Computational Linguistics; 2022. p. 38–49. Available from: `https://aclanthology.org/2022.emnlp-demos.5`.

5. Bertrand M, Kramarz F, Schoar A, Thesmar D. The cost of political connections. Review of Finance. 2018;22(3):849–876.

6. Chen T, Guestrin C. Xgboost: A scalable tree boosting system. In: Proceedings of the 22nd acm sigkdd international conference on knowledge discovery and data mining. ACM; 2016. p. 785–794.

7. Nemacheck CL. Strategic Selection: Presidential Nomination of Supreme Court Justices from Herbert Hoover through George W. Bush. University of Virginia Press; 2007.

8. Black RC, Owens RJ. Courting the president: how circuit court judges alter their behavior for promotion to the Supreme Court. American Journal of Political Science. 2016;60(1):30–43.
