# Supplementary material for: Motivated reasoning in the field: polarization of prose, precedent, and policy in U.S. Circuit Courts, 1891–2013
Source: PLoS One. 2025 Mar 3;20(3):e0318790. doi: 10.1371/journal.pone.0318790 (PMC11875365; doi:10.1371/journal.pone.0318790)
Supplement: Attachment — Submitted filename: Response to Reviewers.pdf [file pone.0318790.s002.pdf]

October 26, 2024

Dear Reviewer #1,

Thank you for your thoughtful and constructive feedback on our manuscript. We greatly appreciate the time and effort you've invested in reviewing our work. Your comments have provided valuable insights that will help us improve the quality and clarity of our paper. We have carefully considered each of your points and would like to address them as follows:

1. **Comment:** *The paper may benefit from a clearer explication of the concept of polarization in this context. Scholars often conceive of it as a reflection of disparate (or diverging) policy preferences. Thus, as a conceptual matter, are the authors using the opinion text as an indicator of polarization, as rooted in judges' preferences? Alternatively, is it more appropriate to conceive of the text-based indicator of polarization as merely the reflection of judges' behavior in each case, which may or may not reflect their sincere preferences?*

   Response:
   Thank you for your insightful feedback on the conceptualization of polarization in our study. We have revised the manuscript to provide a clearer explanation of how we define polarization and its relation to politically motivated reasoning. Specifically, we now define polarization as the observable outcome of politically motivated reasoning, where judges' written opinions and citation patterns reflect ideological biases aligned with their political preferences.

   Revised paragraphs:

   Can we quantitatively identify when judges have an easier time recruiting evidence supporting what they want to be true than evidence supporting what they want to be false Epley and Gilovich (2016)? This tendency is called motivated reasoning, and several recent models and experiments on motivated reasoning are summarized in Bénabou and Tirole (2016). Motivated reasoning is a subject of much policy debate. Does it affect real-world decision-makers? Moreover, what affects motivated reasoning? These are the core questions this paper seeks to address. Motivated reasoning is the well-documented tendency of individuals to actively seek out confirmatory information. The mechanism is said to be implicit emotion regulation, where the brain converges on judgments that maximize positive affective states associated with the attainment of motives. In controlled settings, motivation is typically inferred by the degree to which goal-related concepts are accessible in memory: the greater the motivation, the more likely individuals are to remember, notice, or recognize concepts, objects, or persons related to that goal Touré-Tillery and Fishbach (2014).[1] Recently, motivated reasoning has been used to explain polarization. For example, when responding to moral dilemmas, subjects come to snap judgments and then generate a post hoc justification Haidt (2000); similarly, when interpreting data on climate change, subjects update their beliefs along party lines, particularly among those with higher cognitive reflection Kahan (2013).

   ...

   We analyze over 300,000 Circuit Court opinions (representing almost a million judicial votes) from 1891 to 2013, assessing judicial reasoning through both the text of the opinions and citations to other Circuit Court rulings. These outcomes are linked to judicial biographical factors, particularly political affiliation. In this study, polarization is defined as the degree to which judicial opinions and citation patterns reflect partisan divides, with the reasoning aligning with the judge's political party. We argue that this polarization is an observable result

[1]The classic studies measure the final decision rather than reasoning Zeigarnik (1927); Förster et al. (2005).

of politically motivated reasoning, where judicial reasoning demonstrates ideological biases consistent with the judge's political preferences. Motivated reasoning often skews the decision-making process towards political beliefs, as noted by Thaler (2020), leading judges to unconsciously favor legal interpretations that support their ideological views. This bias results in judges giving more weight to evidence or precedents that align with their pre-existing political beliefs, reinforcing partisan divisions in their legal reasoning. Thus, polarization in judicial opinions can be seen as a direct consequence of motivated reasoning, where cognitive biases lead judges to unconsciously align their reasoning with their political preferences.

The degree of polarization is measured by the accuracy with which we can predict a judge's political affiliation based on the content and structure of their opinions, following the approach of previous studies Gentzkow et al. (2019); Bertrand et al. (2018). Earlier efforts to measure polarization in the text, such as Jensen et al. (2012), might overestimate polarization in early years. Our approach improves upon this by focusing specifically on judicial texts, where political bias can be observed in both language and citation practices. Outside the legal context, Jelveh et al. (2018) use the text of academic articles to predict political donations by economists. Unlike economists or members of Congress, who have discretion over the topics they address, judges are randomly assigned cases. This ex-ante assignment makes judicial reasoning a more direct reflection of political bias. Moreover, political donations are made after (ex-post) economist writings, while the political affiliation of the appointing party is determined before (ex-ante) judicial writings. We seek to predict a predetermined measure of ideology prior to reasoning on the case.

2. **Comment:** *Although the whistleblowing argument (for the analysis of politically divided panels) makes some sense, the argument for expecting less polarization as elections near is not particularly clear. I am not sure why unelected judges would necessarily be all that concerned about the appearance of their opinions right before an election. The public pays little (and often no) attention to lower court judges, absent a few highly publicized cases. So, why should an approaching election make them more sensitive? It seems like any concern for the appearance of an opinion would depend only on the case context.*

   Response:
   Thank you for your thoughtful comments. Regarding your concerns about the argument for reduced polarization as elections approach, it is understandable that unelected judges might not appear to be concerned about public attention. Indeed, as you pointed out, the public often pays little attention to lower court rulings unless they involve highly publicized cases.

   However, the argument for reduced polarization near elections is rooted in the concept of heightened scrutiny during these periods. While public attention may generally be low, there are key stakeholders, such as legal professionals, political actors, and media outlets, who scrutinize judicial behavior more closely around election times, especially when rulings may influence ongoing political debates. This creates an indirect pressure on judges to appear impartial, or at least less overtly partisan, as their decisions could be perceived as having broader political ramifications.

   Moreover, even if judges are not directly elected, the broader political environment in which they operate may lead to a heightened awareness of how their decisions align with party politics, particularly in cases with significant political implications. This scrutiny can be amplified by the political parties that appointed the judges, as these actors may have an interest in how judicial decisions reflect on the party's broader agenda. Therefore, the argument suggests that judges may engage in less polarized reasoning as a strategy to avoid potential backlash or accusations of partisanship, particularly during high-stakes periods like elections.

   Finally, as the analysis in the paper indicates, the effect of approaching elections on polarization is not uniform

across all judges or cases. Judges facing promotion incentives, or those being considered for higher courts, may be more sensitive to the appearance of impartiality, especially in cases where their rulings could attract attention from influential legal or political actors.

Added paragraph:

The argument for reduced polarization near elections hinges on the heightened scrutiny that judges face during these periods. Key stakeholders—such as political actors, legal professionals, and the media—closely observe judicial decisions, especially when those decisions could have broader political ramifications. This scrutiny creates indirect pressure on judges to appear impartial or less overtly partisan, even if they are not directly elected. Moreover, the political environment in which judges operate may heighten their awareness of how their rulings align with party politics. As a result, judges may strategically temper their reasoning to avoid potential accusations of partisanship or political backlash, particularly during high-stakes periods like elections. This tendency helps explain the reduction in polarization we observe in judicial opinions and citations, particularly leading up to midterm elections, when such scrutiny may be most intense.

3. **Comment:** *The authors make several potentially problematic assumptions that they might want to further defend/explore. First, they emphasize the randomness of panel selection in the U.S. Courts of Appeals as an essential foundation for their research design. However, some research has questioned whether panel randomness exists in practice – see, e.g., Levy & Chilton 2015 and Levy 2020 (Cornell Law Review). And, it may be that non-randomness is more prevalent in certain circuits (e.g., the Ninth Circuit). The authors briefly note this possibility in footnote* #6, *but they ought to address it earlier (e.g., they feature random panel selection at the beginning of the paper, only to hedge and call it "quasi-random" several pages later).*

   Also, while the authors argue that political party appears unrelated to panel assignment, it could be that certain judges exhibit nonrandom selection based on the policy issue, which might correlate with partisanship. Thus, I think the authors should do more to scrutinize the assumption that any nonrandom selection is unrelated to party.

   Next, the authors focus on opinions "written by one" judge while dropping per curiam opinions and those they claim to identify as "drafted by multiple judges." The author's key assumption is that they can attribute the opinion text to just a single judge. However, a substantial literature shows that bargaining over opinion content and panel effects influence U.S. Courts of Appeals decisions. Thus, there are within-panel dynamics where it is possible, if not likely, that multiple judges leave their fingerprints on the majority opinion.

   Also, the authors assume that all parties bring all relevant arguments to the court's attention in their briefs. I am not sure that this is necessarily the case, especially given there is reason for the parties to strategically select/push particular arguments. Perhaps the two parties combined will cover the full terrain in the end. But, given the evidence (and reason) to expect strategic attorneys, I have some doubt about the authors' contention that the parties feel obligated to cover all the argument bases.

   Response:
   Thank you for your thoughtful feedback.

   We acknowledge the importance of panel randomness as a foundation for this research design. To address concerns around non-random assignment, we have performed robustness checks by excluding the Second, Eighth, Ninth, and D.C. Circuits, which previous research identifies as potentially less random in panel assignments (Chilton and Levy, 2015). Our findings remain consistent even after these exclusions, shown in Table 1 and 2.

Furthermore, Chen et al. (2016) provides evidence suggesting that panel compositions do not exhibit time-based autocorrelation, further reinforcing the validity of this assumption.

Table 1: Polarization in Divided Panels (Dropping Court 2, 8, 9, and DC)

	(1) Text	(2) Citation	(3) Dissent vote
Divided Panel	-0.034***	-0.041***	0.004***
	(0.007)	(0.005)	(0.001)
Observations	178396	154920	598232
R^2	0.306	0.123	0.011
Circuit × Year FE	✓	✓	✓
Legal Issue FE	✓	✓	✓

Notes: This table shows how judges on a divided panel would exhibit polarization in prose, precedent, and policy. The unit of observation for Column (1) and (2) is at the opinion level, and Column (3) is at the vote level. Every case has three votes from three judges sitting in a panel and judges are allowed to write concurring or dissent opinions besides the majority opinion for each case. We controlled for Circuit × Year and legal issues fixed effects. Standard errors clustered at judge level in parentheses.* $p < 0.1$,** $p < 0.05$,*** $p < 0.01$

Table 2: Polarization in Divided Panels (Dropping Court 2, 8, 9, and DC)

	Text (1)	Citation (2)	Dissent Vote (3)
Minority	-0.018**	-0.030***	0.009***
	(0.008)	(0.005)	(0.001)
Observations	129135	112337	426877
R^2	0.295	0.068	0.013
Circuit × Year FE	✓	✓	✓
Legal Issue FE	✓	✓	✓

Notes: Effect of being a minority judge (D of DRR or R of RDD) on the polarization in texts and citations, and the likelihood to cast a dissenting vote, controlling for Circuit × Year and legal issues fixed effects. The unit of observation for Column (1) and (2) is at the opinion level, and Column (3) is at the vote level. Standard errors clustered at judge level in parentheses. The sample is cases with judges from both political parties. $*p < .1, **\ p < 0.05, ***p < .01$.

While we assert that political party affiliation generally does not drive panel assignment, we recognize that case type might indirectly correlate with judges' partisan affiliations. However, existing research shows that circuit judges' personal characteristics, including political appointment, do not correlate strongly with case characteristics or the partisan composition of other panel members (Chen et al., 2023). Thus, while case types may vary, we find no compelling evidence that selection within panels systematically aligns with party politics.

We acknowledge that within-panel dynamics could influence opinion content. However, we conduct robustness checks focused on senior judges who oversee opinion assignments to account for potential multi-judge influence, shown in Table 3 and 4. Our results remain consistent, supporting our assumption that we can attribute opinion text predominantly to a single judge. We also included these tables and our discussion here in the manuscript as a separate paragraph.

The assumption that litigants present all relevant bases for persuasive arguments remains justified, even when strategic behavior occurs. Litigants are incentivized to bring forth persuasive arguments. Although knowledge of the panel composition could potentially affect the emphasis of certain arguments, they must file briefs before judges are assigned, which limits the ability to strategically tailor arguments based on specific judges. Indeed, one study finds that when judges are revealed earlier to the lawyers, there is no effect on the likelihood of settlement, a concrete form of strategic behavior that appears minimal in this context.

Added paragraph:

To address concerns around non-random assignment, we have performed robustness checks by excluding the Second, Eighth, Ninth, and D.C. Circuits, which previous research identifies as potentially less random in panel assignments (Chilton and Levy, 2015). Our findings remain consistent even after these exclusions, shown in Table 1 and 2. Furthermore, Chen et al. (2016) provides evidence suggesting that panel compositions do not exhibit time-based autocorrelation, further reinforcing the validity of this assumption. We are also aware that within-panel dynamics could influence opinion content. However, we conduct robustness checks focused on senior judges who oversee opinion assignments to account for potential multi-judge influence, shown in Table 3 and 4. Our results remain consistent, supporting our assumption that we can attribute opinion text predominantly to a single judge.

Table 3: Polarization in Divided Panels (Senior judges)

	(1)<br>Text	(2)<br>Citation	(3)<br>Dissent vote
Divided Panel	-0.046***	-0.047***	0.007***
	(0.007)	(0.005)	(0.002)
Observations	100726	87842	327994
R^2	0.356	0.206	0.014
Circuit × Year FE	✓	✓	✓
Legal Issue FE	✓	✓	✓

Notes: This table shows how judges on a divided panel would exhibit polarization in prose, precedent, and policy. The unit of observation for Column (1) and (2) is at the opinion level, and Column (3) is at the vote level. Every case has three votes from three judges sitting in a panel and judges are allowed to write concurring or dissent opinions besides the majority opinion for each case. We controlled for Circuit × Year and legal issues fixed effects. Standard errors clustered at judge level in parentheses.* $p < 0.1$,** $p < 0.05$,*** $p < 0.01$

Table 4: Polarization in Divided Panels (Senior judges)

	Text	Citation	Dissent Vote
	(1)	(2)	(3)
Minority	-0.036***	-0.045***	0.016***
	(0.008)	(0.005)	(0.002)
Observations	100726	87842	327994
R^2	0.354	0.207	0.015
Circuit × Year FE	✓	✓	✓
Legal Issue FE	✓	✓	✓

Notes: Effect of being a minority judge (D of DRR or R of RDD) on the polarization in texts and citations, and the likelihood to cast a dissenting vote, controlling for Circuit × Year and legal issues fixed effects. The unit of observation for Column (1) and (2) is at the opinion level, and Column (3) is at the vote level. Standard errors clustered at judge level in parentheses. The sample is cases with judges from both political parties. $*p < .1, **p < 0.05, ***p < .01$.

The assumption that parties bring all relevant arguments to the court remains justified, even accounting for potential strategic prioritization. Attorneys are bound by ethical standards and professional incentives to present all essential arguments to avoid exploitable gaps. Strategic elements, such as panel composition knowledge, may guide emphasis but do not imply omission of key points. This adaptive approach aligns with their obligation to advocate fully, maintaining the robustness of the comprehensive argumentation assumption even within strategic frameworks (Jordan, 2007).

4. **Comment:** *The authors give no consideration to the role of law clerks in drafting opinions. Is there consistency in polarization across a judge's tenure? Some literature has used a lack of opinion text consistency over time as an indication that clerks may be more involved in drafting opinions. Of course, there may be other reasons why judges' opinion text changes over time. But, as it stands now, I see no indication that the authors have considered how law clerks might undermine their research design and empirical results.*

   Response:
   Thank you for raising this important point. While we agree that law clerks play a significant role in drafting opinions, we believe that this does not undermine our research design or empirical results. Judges, as you noted, choose their clerks, and this selection process is often highly influenced by the judge's own ideological preferences. As a result, clerks are likely to share or at least align with the judge's legal and political views, which ensures a consistency in the ideological slant of the opinions over time.

   Moreover, the fact that judges have full discretion over the hiring of their clerks suggests that the influence of clerks on opinion-writing is not independent of the judge's own biases and motivations. Judges can actively shape their opinions by selecting clerks who reflect their judicial philosophy. This helps to maintain consistency in the tone and reasoning of judicial decisions throughout a judge's tenure.

   That said, we recognize that shifts in writing style or reasoning might occur over time, and this could be due to various factors, including clerk involvement. However, we argue that any potential variation introduced by clerks is itself a reflection of the judge's preferences and should not be seen as an external distortion of the research design. The overarching ideological orientation, which is our focus, remains primarily driven by the judge, even if clerks contribute to the drafting process.

5. **Comment:** *The paper would benefit from more substantive explanation of how the authors' measurement approach translates text into an indicator of polarization. For instance, explain in less technical terms how the machine-learning approach yields a measure that meaningfully conveys judges' propensity to use politically motivated language in their opinions.*

   Response:
   Thank you for this valuable suggestion. We agree that a clearer explanation of our measurement approach would enhance the paper's accessibility and impact.

   To address this, we modified the related paragraph in our methodology. This addition includes:

   - Provide a conceptual overview of how our machine learning approach 'learns' to identify politically charged language.
   - Explain in simple terms how our polarization measure reflects a judge's propensity to use politically motivated language.

   Revised paragraph:

   In less technical terms, we "train" the machine-learning model on a large set of texts where we already know the judge's political affiliation. The model then learns which linguistic features (words, phrases, types of arguments, etc.) are more frequently used by judges from each political party. Once trained, the model can take a new judicial opinion, analyze its language, and estimate how likely the judge is to align with a particular political ideology based on the patterns in their writing.

   This measure of polarization reflects how much the language in an opinion resembles the typical language used by judges of a specific political party. If the opinion strongly mirrors the patterns associated with one party, we interpret this as evidence of politically motivated reasoning. Conversely, if the opinion uses language that doesn't clearly align with any political party, it would suggest a lower degree of polarization.

6. **Comment:** *Perhaps I missed it, but it's not clear to me why the authors do not use concurring opinions in their analysis.*

   Response:
   Thank you for this insightful observation. Our analysis centers on majority and dissenting opinions, as these capture the primary judicial reasoning and ideological divides. The decision to dissent and concur—and the content of that concurrence—is itself a choice by the judge, potentially influenced by personal or contextual factors unrelated to the core decision-making process. Similar to these decisions, research about Congressional speech (Gentzkow et al., 2019), show that congressmen choose both to speak and what to say. In our study, we aimed to leverage the random assignment and "forced" nature of majority and dissenting opinions to isolate core judicial perspectives without the self-selection that accompanies concurrences, introducing additional endogneity.

7. **Comment:** *The paper would benefit (if space permits) from a bit more background theoretical discussion of why judges (as humans) engage in motivated reasoning.*

   Response:
   We appreciate the reviewer's suggestion to expand on the theoretical background of motivated reasoning in the context of judicial decision-making. In response, we have added a paragraph that draws on Thaler (2020)'s framework of politically motivated reasoning. This addition explains how motivated reasoning operates as a cognitive process where individuals, including judges, distort information to align with their pre-existing beliefs, often to reduce cognitive dissonance. We further clarify how this framework applies to judicial reasoning, illustrating

how political affiliation can unconsciously influence how judges interpret legal precedents and facts. The revised discussion provides a more robust theoretical foundation for understanding why judges, like all individuals, may engage in motivated reasoning, which contributes to the observed ideological polarization in judicial opinions.

Added paragraph:

Motivated reasoning can be understood as a cognitive process where individuals distort how they interpret information to align with their pre-existing beliefs, often to reduce cognitive dissonance. Thaler (2020)'s framework on politically motivated reasoning provides a clear explanation of how and why this occurs, especially in politically charged contexts. His experimental work shows that individuals do not update their beliefs in a purely Bayesian manner when receiving new information. Instead, they give greater weight to information that reinforces their prior beliefs and are more skeptical of information that challenges those beliefs. Applied to judges, this framework helps explain why judicial reasoning can exhibit ideological polarization. Even though judges are expected to be impartial, their political affiliation and identity can lead them to unconsciously distort how they interpret legal precedents or facts, much like how Thaler's subjects over-trusted news that supported their political stances. In legal contexts, where ambiguity often exists, this motivated reasoning can manifest as judges unconsciously favoring interpretations of the law that align with their political preferences. Thaler's model also predicts that the stronger an individual's prior beliefs (such as a judge's political affiliation), the more likely they are to exhibit overconfidence in the correctness of their reasoning, further reinforcing polarized judicial opinions.

Once again, we sincerely thank you for your insightful comments and suggestions. Your feedback has been invaluable in helping us identify areas for improvement in our manuscript. We believe that addressing these points will significantly strengthen our paper and provide a more comprehensive analysis of judicial polarization.

Sincerely,

Dr. Wei Lu
Assistant Professor of Marketing
CUNY–Baruch College
55 Lexington Ave, New York, NY 10010
wei.lu@baruch.cuny.edu

Dr. Daniel L. Chen
Professor
Toulouse School of Economics
Institute for Advanced Study at Toulouse
21 allée de Brienne, Toulouse, 31015, France
dlchen@nber.org

October 26, 2024

Dear Reviewer #2,

Thank you for your thoughtful and constructive feedback on our manuscript. We sincerely appreciate the time and effort you've dedicated to reviewing our work. Your comments have offered valuable insights that will greatly enhance the quality and clarity of our paper. We have carefully considered each of your suggestions and would like to address them in detail as follows:

1. **Comment:** *First, I found the manuscript lacking in its theoretical grounding around motivated reasoning. The authors introduce the concept, but they fail to establish a clear link between motivated reasoning and judicial behavior, to say nothing of the link between motivated reasoning, judicial behavior, and political partisanship. Indeed, by the latter half of the paper, much of the discussion of motivated reasoning is gone, and the discussion entirely centers on polarization, but the genesis of the switch between motivated reasoning to polarization is never made clear. Simply asserting that judges with different political orientations reason differently doesn't connect well with the psychological literature around motivated reasoning. A more detailed exploration of motivated reasoning, and how it connects here–including consideration of things like cognitive biases–would substantially improve this. In all, I found the theoretical framing not to be a compelling link.*

   Response:
   We would like to thank the reviewer for their insightful feedback, which has helped us improve the clarity and theoretical grounding of our manuscript. Below, we outline how we have addressed the concerns raised.

   1. Theoretical Grounding of Motivated Reasoning

   The reviewer expressed concern about the lack of theoretical grounding around motivated reasoning and its connection to judicial behavior. In response, we have revised the relevant sections to provide a clearer link between motivated reasoning and judicial decision-making. Specifically, we now emphasize how motivated reasoning operates through cognitive biases, such as confirmation bias, which leads judges to unconsciously favor legal interpretations that align with their political preferences. This revision aligns our discussion with relevant psychological literature and strengthens the connection between motivated reasoning and judicial behavior.

   2. Link Between Motivated Reasoning, Judicial Behavior, and Political Partisanship

   We acknowledge the reviewer's concern regarding the missing link between motivated reasoning, judicial behavior, and political partisanship. In the revised manuscript, we explicitly define polarization as the observable outcome of politically motivated reasoning. We argue that motivated reasoning distorts the updating process in favor of political beliefs, leading to partisan divisions in judicial opinions. The revised text further explains that even under conditions of random case assignment, judges' reasoning aligns with their political affiliation, demonstrating how motivated reasoning influences judicial behavior. This addresses the reviewer's request for a more robust theoretical framing of these connections.

   3. Clarifying the Shift from Motivated Reasoning to Polarization

   In response to the reviewer's observation about the transition from motivated reasoning to polarization, we have revised the manuscript to clarify that polarization in judicial opinions is a direct consequence of motivated reasoning. By elaborating on how cognitive biases shape judicial reasoning and result in ideological divisions, we provide a clearer and more cohesive explanation of how these concepts are linked within the framework of our study.

Revised paragraphs:

We analyze over 300,000 Circuit Court opinions (representing almost a million judicial votes) from 1891 to 2013, assessing judicial reasoning through both the text of the opinions and citations to other Circuit Court rulings. These outcomes are linked to judicial biographical factors, particularly political affiliation. In this study, polarization is defined as the degree to which judicial opinions and citation patterns reflect partisan divides, with the reasoning aligning with the judge's political party. We argue that this polarization is an observable result of politically motivated reasoning, where judicial reasoning demonstrates ideological biases consistent with the judge's political preferences. Motivated reasoning often skews the decision-making process towards political beliefs, as noted by Thaler (2020), leading judges to unconsciously favor legal interpretations that support their ideological views. This bias results in judges giving more weight to evidence or precedents that align with their pre-existing political beliefs, reinforcing partisan divisions in their legal reasoning. Thus, polarization in judicial opinions can be seen as a direct consequence of motivated reasoning, where cognitive biases lead judges to unconsciously align their reasoning with their political preferences.

The degree of polarization is measured by the accuracy with which we can predict a judge's political affiliation based on the content and structure of their opinions, following the approach of previous studies Gentzkow et al. (2019); Bertrand et al. (2018). Earlier efforts to measure polarization in the text, such as Jensen et al. (2012), might overestimate polarization in early years. Our approach improves upon this by focusing specifically on judicial texts, where political bias can be observed in both language and citation practices. Outside the legal context, Jelveh et al. (2018) use the text of academic articles to predict political donations by economists. Unlike economists or members of Congress, who have discretion over the topics they address, judges are randomly assigned cases. This ex-ante assignment makes judicial reasoning a more direct reflection of political bias. Moreover, political donations are made after (ex-post) economist writings, while the political affiliation of the appointing party is determined before (ex-ante) judicial writings. We seek to predict a predetermined measure of ideology prior to reasoning on the case.

2. **Comment:** *Second, and related, the lack of a cohesive theoretical framework compounds the challenges for the authors in measurement and in interpreting their empirical results. On the measurement side, capturing motivated reasoning as the extent to which party affiliation is predictable by language requires significantly more justification; shifting that to then be an indicator for political polarization requires yet more. On the empirical results, the lack of a theoretical framework and the underjustified measurement create a lot of uncertainty for interpretation. For instance, for divided panels, the authors find that polarization in texts and citations is lower, but that judges sitting on these divided panels are more likely to dissent. There's no real story around why any of this might be the case. The authors note a general idea about heightened scrutiny on divided panels, but the explanation is not sufficiently developed from the earlier theoretical discussions. Moreover, why is the dissent separate at all? If the idea is that divided panels encourage scrutiny, and this precipitates each of these changes in polarization, shouldn't the dissent be another component of scrutiny? The authors could improve this, again, by building a real theoretical framework from motivated reasoning, but at present that is absent.*

   Response:

   Revised paragraphs:

   Our investigation begins with how judicial reasoning and decisions are influenced when judges serve on a three-member panel that consists of both Republican and Democratic appointees. According to Thaler (2020)'s framework, motivated reasoning occurs when individuals process information in a way that aligns with their prior beliefs, especially in politically charged environments. In judicial settings, motivated reasoning would predict

that judges are likely to favor legal interpretations that support their political ideology. However, when judges are part of politically divided panels, they face heightened scrutiny from colleagues with opposing ideological views. This scrutiny leads to two distinct but connected effects: it moderates how judges express their reasoning in written opinions, but it does not eliminate ideological disagreements, which manifest as dissenting votes.

Judges under scrutiny may temper their written opinions to avoid overt expressions of partisanship that could be easily criticized by opposing panel members. This occurs because the diverse panel composition forces judges to justify their legal reasoning in a way that can withstand scrutiny from colleagues with different ideological viewpoints. As a result, we observe a reduction in polarization in the language and legal citations of their opinions.

However, while motivated reasoning may be constrained in the written text, ideological differences still exist at a deeper level. These differences are harder to suppress in the final decision-making process, which often results in dissenting votes. Dissent, in this case, becomes a way for judges to express their underlying political beliefs when they cannot fully align with the majority opinion. Voting behavior, unlike written opinions, is less subject to the same degree of scrutiny because it is a direct reflection of each judge's individual stance on the case outcome. Therefore, even though judges moderate their reasoning to align with doctrinal standards under scrutiny, they are still likely to cast dissenting votes when their core ideological beliefs diverge from the majority. Moreover, previous studies indicate that political divisions within a panel creates an opportunity for whistleblowing, through dissenting opinions, to expose disobedient decision-making by the majority. In the presence of such a whistleblower, the majority must sometimes capitulate and keep its decision within the confines of doctrine Cross and Tiller (1997); Beim et al. (2021); Cohen (2023).

To explore these dynamics, we employ a linear regression model that examines polarization in judicial reasoning and dissenting votes, with the key independent variable being participation in a politically divided panel. The counterfactual group consists of judges participating in politically homogeneous panels. This model controls for Circuit × Year and legal issues fixed effects. In Table 2, we also look at the scenario of whether a judge sitting on a divided panel is a minority judge in terms of political affiliations. In this situation, heightened scrutiny will lead to even greater moderation in reasoning, considering the composition of the panel.

3. **Comment:** *Finally, the data here are impressive. However, the authors seem to really miss an opportunity to explore the over-time institutional and political transformations that occurred within the federal courts of appeals over this period. Indeed, there are major works in political science and legal studies on the changing partisan composition of the federal courts over this time period. Because the focus is on changing polarization, the absence of these discussions felt particularly pronounced. How do the observed changes match with the changing institutional parameters of the federal courts of appeals? Are there institutional predicates for the observed changes in polarization? Without engaging with these developments, the analysis holds almost entirely on an undertheorized story around individual judges polarizing.*

   Response:
   Thank you for this insightful comment. We agree that incorporating the institutional and political transformations of federal courts of appeals would provide valuable context for understanding the patterns of polarization we observe. The rich literature on changing partisan composition and institutional development of circuit courts could indeed help explain some of our findings.

   While our paper focuses primarily on measuring and documenting patterns of motivated reasoning through text analysis and citation networks, we acknowledge that a deeper engagement with institutional changes would strengthen the theoretical foundations of our work. Future research could productively examine how specific

institutional reforms, changes in appointment processes, and shifting partisan dynamics shaped the patterns we observe, such as deviations from mainstream ideology (Chen et al., 2023), promotion incentives (Epstein and Segal, 2005), etc.

Given space constraints and our focus on developing novel empirical measures of judicial reasoning, we chose to focus primarily on documenting and analyzing polarization patterns. However, we agree that future work integrating our empirical approach with institutional analysis would be valuable for understanding the deeper causes of judicial polarization. We have added a note acknowledging this limitation and suggesting it as an important direction for future research.

Once again, we sincerely thank you for your insightful comments and suggestions. Your feedback has been invaluable in helping us refine key areas of our manuscript. We are confident that addressing these points will greatly enhance the strength of our paper and contribute to a more comprehensive analysis of judicial polarization.

Sincerely,

Dr. Wei Lu
Assistant Professor of Marketing
CUNY–Baruch College
55 Lexington Ave, New York, NY 10010
wei.lu@baruch.cuny.edu

Correspondent Author: Dr. Daniel L. Chen
Professor
Toulouse School of Economics, Institute for Advanced Study at Toulouse
21 allée de Brienne, Toulouse, 31015, France
dlchen@nber.org

References

Beim, Deborah, Tom S Clark, and Benjamin E Lauderdale, "Republican-majority appellate panels increase execution rates for capital defendants," *The Journal of Politics*, 2021, *83* (3), 1163–1167. 11

Bénabou, Roland and Jean Tirole, "Mindful economics: The production, consumption, and value of beliefs," *Journal of Economic Perspectives*, 2016, *30* (3), 141–64. 1

Bertrand, Marianne, Francis Kramarz, Antoinette Schoar, and David Thesmar, "The cost of political connections," *Review of Finance*, 2018, *22* (3), 849–876. 2, 10

Chen, Daniel L, Moti Michaeli, and Daniel Spiro, "Non-confrontational extremists," *European Economic Review*, 2023, *157*, 104521. 4, 12

_ , Vardges Levonyan, and Susan Yeh, "Can policies affect preferences: evidence from random variation in abortion jurisprudence," *TSE Working Paper*, 2016. 4, 5

Chilton, Adam S and Marin K Levy, "Challenging the Randomness of Panel Assignment in the Federal Courts of Appeals," *Cornell L. Rev.*, 2015, *101*, 1. 3, 5

Cohen, Alma, "The Pervasive Influence of Ideology at the Federal Circuit Courts," Technical Report, National Bureau of Economic Research 2023. 11

Cross, Frank B and Emerson H Tiller, "Judicial partisanship and obedience to legal doctrine: Whistleblowing on the federal courts of appeals," *Yale LJ*, 1997, *107*, 2155. 11

Epley, Nicholas and Thomas Gilovich, "The mechanics of motivated reasoning," *Journal of Economic perspectives*, 2016, *30* (3), 133–40. 1

Epstein, Lee and Jeffrey A Segal, *Advice and consent: The politics of judicial appointments*, Oxford University Press, 2005. 12

Förster, Jens, Nira Liberman, and E Tory Higgins, "Accessibility from active and fulfilled goals," *Journal of Experimental Social Psychology*, 2005, *41* (3), 220–239. 1

Gentzkow, Matthew, Jesse M Shapiro, and Matt Taddy, "Measuring Group Differences in High-Dimensional Choices: Method and Application to Congressional Speech," *Econometrica*, 2019, *87* (4), 1307–1340. 2, 7, 10

Haidt, Jonathan, "The Positive emotion of elevation.," *Prevention & Treatment*, 2000, *3* (1). 1

Jelveh, Zubin, Bruce Kogut, and Suresh Naidu, "Political language in economics," Technical Report 14-57 2018. 2, 10

Jensen, Jacob, Suresh Naidu, Ethan Kaplan, Laurence Wilse-Samson, David Gergen, Michael Zuckerman, and Arthur Spirling, "Political polarization and the dynamics of political language: Evidence from 130 years of partisan speech [with comments and discussion]," *Brookings Papers on Economic Activity*, 2012, pp. 1–81. 2, 10

Jordan, Samuel P, "Early Panel Announcement, Settlement, and Adjudication," *BYU L. Rev.*, 2007, p. 55. 6

Kahan, Dan M, "Ideology, motivated reasoning, and cognitive reflection," *Judgment and Decision Making*, 2013, *8* (4), 407–424. 1

Thaler, Michael, "The fake news effect: Experimentally identifying motivated reasoning using trust in news," *arXiv preprint arXiv:2012.01663*, 2020. 2, 7, 8, 10

Touré-Tillery, Maferima and Ayelet Fishbach, "How to measure motivation: A guide for the experimental social psychologist," *Social and Personality Psychology Compass*, 2014, *8* (7), 328–341. 1

Zeigarnik, Bluma, "On the retention of completed and uncompleted activities," *Psychologische Forschung*, 1927, *9*,

1–85. 1
