# Supplementary material for: Motivated reasoning in the field: polarization of prose, precedent, and policy in U.S. Circuit Courts, 1891–2013
Source: PLoS One. 2025 Mar 3;20(3):e0318790. doi: 10.1371/journal.pone.0318790 (PMC11875365; doi:10.1371/journal.pone.0318790)
Supplement: Attachment — Submitted filename: response_to_reviewers.pdf [file pone.0318790.s003.pdf]

January 16, 2025

Dear Reviewer #1,

Thank you for your thoughtful and constructive feedback on our manuscript. We greatly appreciate the time and effort you've invested in reviewing our work. Your comments have provided valuable insights that will help us improve the quality and clarity of our paper. We have carefully considered each of your points and would like to address them as follows:

1. **Comment:** *I appreciate the authors' engagement with my original review. On balance, I am satisfied with their responses and revisions. I think they have meaningfully improved the paper and thus I recommend publication. However, in my view, there is one critique/response that would still benefit from more attention from the authors.*

   I think the authors' argument related to election-year effects still lacks a clear theoretical mechanism. The central argument that judges want to appear less partisan or avoid attracting scrutiny makes some sense on its face, but I don't see any discussion of why judges should care. That is, why do unelected judges with life tenure care about appearing less partisan or avoiding scrutiny? I think the conditional effect for judges facing promotion incentives makes some sense and is a partial answer. But, it still doesn't fully address the question of why the average circuit judge, on balance, cares about avoiding scrutiny. And, furthermore, I am still skeptical that anyone cares all that much about scrutinizing many of these cases (i.e., perhaps this argument and empirical effect ought to be entirely conditional on certain salient cases). So, while I appreciate the authors' engagement on this point, I still have some questions about this original critique.

   Response:
   We appreciate the referee's request for additional development regarding why judges care about heightened scrutiny. In brief, we argue that a judge's concern for reputation, the possibility of "whistleblowing" by colleagues, fear of reversal, and career advancement considerations can all drive judges to moderate the polarization in their opinions when scrutiny increases. Below we elaborate and point to relevant evidence (both quantitative and qualitative) and complementary literature.

   (a) Reputation and Collegial Dynamics.

   - Whistleblowing: As Cross and Tiller (1997) argue, having even one judge on the panel from the opposing political party (or an ideologically different judge) increases the risk of dissent or "whistleblowing." A public dissent can highlight legal or doctrinal shortcomings in a majority opinion, thus putting the majority opinion's author "on alert" that the opinion must withstand potentially negative attention from colleagues, higher courts, and the broader legal community.
   - Professional Standing: Judges in appellate courts typically belong to tight-knit legal communities. The desire to maintain respect among peers (within the judiciary and beyond) can temper overt expressions of political bias, especially when one's reasoning might be called out immediately by a panel colleague or attract critical media coverage.

   (b) Fear of Reversal and Adverse Consequences.

   - Higher-Court Review: While U.S. Circuit Court judges enjoy considerable autonomy, they still stand beneath the Supreme Court. Even if reversal is statistically rare, a published opinion that is overtly partisan or that strays too far from precedent can draw the notice of higher courts. Circuit judges typically strive to avoid any damaging signals that their reasoning is outcome-driven rather than rooted

in legal principles (see Hasen (2019) for a review of how higher courts, particularly the Supreme Court, can spotlight ideological extremes).

- Reputation in Legal Scholarship & Media: The fear of having an opinion singled out as unreasoned or obviously partisan—particularly in high-profile cases—can impose a reputation cost. Even if not formally "reversed," a judge can be criticized in law reviews, mainstream media, or professional bodies (bar associations, judicial councils).

(c) Career Advancement Incentives.

- Potential for Promotion: Judges looking ahead to a possible Supreme Court nomination (or elevated administrative roles within the judiciary) might choose to signal "mainstream" credibility. Black and Owens (2016) find that judges who appear on shortlists for promotion sometimes alter their judicial behavior (e.g., by writing more separate opinions). Although that evidence focuses on dissent votes, it parallels our reasoning regarding the rhetorical strategy and precedent selection that judges adopt when they know their decisions and opinions might be particularly visible to influential observers in Washington.
- Political Actors' Influence: Presidential or Senate interest in a particular judge's record can further heighten scrutiny—especially near election cycles or when there are rumors of a Supreme Court vacancy. This dynamic is recognized in work by Berdejó and Chen (2017) and Chen (2024), who show that judges can strategically moderate or adapt their behavior (e.g., in sentencing or other policy domains) in politically sensitive time windows.

(d) Empirical and Qualitative Evidence Supporting These Mechanisms.

- Empirical Support: Numerous studies (e.g., Beim et al. (2021); Cohen (2023); Choi and Gulati (2008)) document how judges' voting and opinion-writing patterns shift in contexts where an "opposing" voice on the panel might expose deviations from established doctrine. In our own data, we observe that judges moderate their textual or citation polarization when facing a divided panel (see Tables 1 and 2).
- Qualitative/Interview Evidence: While direct interviews with circuit judges can be challenging to conduct, there is anecdotal evidence—such as public statements by retired judges or academic commentary—suggesting that professional pride, personal relationships with fellow judges, and media attention can influence how opinions are drafted. Judge Richard Posner's numerous interviews and writings (e.g., New York Times, September 11, 2017 interview) illustrate that some judges are aware of, and candid about, the rhetorical choices they make in published opinions.

In sum, judges do not operate in a vacuum. Collegial interactions, fear of reputation damage, and a desire to avoid censure—by dissenting colleagues, higher courts, political actors, or the media—can make judges care about heightened scrutiny. These factors give judges strong incentives to craft their language and select precedents in ways that minimize overtly partisan cues, even if their underlying preferences remain the same. Our findings that judges display less polarization in their written reasoning under scrutiny (but not necessarily in their voting) are consistent with this logic of reputational and career-related concerns.

Revised paragraphs:

Although U.S. Circuit Court judges enjoy life tenure, they do not operate in a vacuum. The argument for reduced polarization near elections hinges on the heightened scrutiny that judges face during these periods. Key stakeholders—such as political actors, legal professionals, and the media—pay particularly close attention to judicial decisions with potential political ramifications, creating indirect pressure on judges to appear impartial or

less overtly partisan Choi and Gulati (2008). This scrutiny may heighten judges' awareness of how their rulings align with party politics, prompting them to strategically temper their written reasoning to avoid accusations of partisanship or backlash—especially in high-stakes election contexts Cross and Tiller (1997); Hasen (2019). Even the presence of a single judge from the opposing party on a panel can lead to "whistleblowing" through dissent, raising the reputational stakes in a close-knit appellate community. Furthermore, although reversal by the Supreme Court remains statistically rare, judges seek to avoid decisions that might appear excessively outcome-driven and invite negative attention Hasen (2019). Career advancement incentives add another layer: Black and Owens (2016) find that judges who appear on shortlists for promotion sometimes alter their judicial behavior, a pattern that parallels evidence of electoral-cycle adjustments in other judicial domains Berdejó and Chen (2017); Chen (2016). More broadly, judges' concerns about professional standing, institutional legitimacy, and adherence to norms of impartiality Kahan (2016); Beim et al. (2021); Cohen (2023) can all contribute to a moderation in overt partisanship near elections, helping explain the decline in polarization we observe in both opinion text and citations leading up to midterm contests.

Once again, we sincerely thank you for your insightful comment. Your feedback has been invaluable in helping us identify areas for improvement in our manuscript. We believe that addressing these points will significantly strengthen our paper and provide a more comprehensive analysis of judicial polarization.

Sincerely,

Dr. Wei Lu
Assistant Professor of Marketing
CUNY–Baruch College
55 Lexington Ave, New York, NY 10010
wei.lu@baruch.cuny.edu

Dr. Daniel L. Chen
Professor
Toulouse School of Economics
Institute for Advanced Study at Toulouse
21 allée de Brienne, Toulouse, 31015, France
dlchen@nber.org

References

Beim, Deborah, Tom S Clark, and Benjamin E Lauderdale, "Republican-majority appellate panels increase execution rates for capital defendants," *The Journal of Politics*, 2021, *83* (3), 1163–1167. 2, 3

Berdejó, Carlos and Daniel L Chen, "Electoral cycles among us courts of appeals judges," *The Journal of Law and Economics*, 2017, *60* (3), 479–496. 2, 3

Black, Ryan C and Ryan J Owens, "Courting the president: how circuit court judges alter their behavior for promotion to the Supreme Court," *American Journal of Political Science*, 2016, *60* (1), 30–43. 2, 3

Chen, Daniel L, "Priming Ideology: Why Presidential Elections Affect US Judges," Technical Report, Institute for Advanced Study in Toulouse (IAST) 2016. 3

_ , "Priming ideology I: Why do presidential elections affect US judges," *European Economic Review*, 2024, *169*, 104835. 2

Choi, Stephen J and G Mitu Gulati, "Bias in judicial citations: a window into the behavior of judges?," *The Journal of Legal Studies*, 2008, *37* (1), 87–129. 2

Cohen, Alma, "The Pervasive Influence of Ideology at the Federal Circuit Courts," Technical Report, National Bureau of Economic Research 2023. 2, 3

Cross, Frank B and Emerson H Tiller, "Judicial partisanship and obedience to legal doctrine: Whistleblowing on the federal courts of appeals," *Yale LJ*, 1997, *107*, 2155. 1, 3

Hasen, Richard L, "Polarization and the Judiciary," *Annual Review of Political Science*, 2019, *22*, 261–276. 2, 3

Kahan, Dan M, "The politically motivated reasoning paradigm, part 1: What politically motivated reasoning is and how to measure it," *Emerging trends in the social and behavioral sciences: An interdisciplinary, searchable, and linkable resource*, 2016, pp. 1–16. 3
